# *In Vitro* Screening for Abiotic Stress Tolerance in Potent Biocontrol and Plant Growth Promoting Strains of *Pseudomonas* and *Bacillus* spp.

**DOI:** 10.1155/2014/195946

**Published:** 2014-03-06

**Authors:** G. Praveen Kumar, S. K. Mir Hassan Ahmed, Suseelendra Desai, E. Leo Daniel Amalraj, Abdul Rasul

**Affiliations:** Division of Crop Sciences, Central Research Institute for Dryland Agriculture, Santoshnagar, Saidabad Post, Hyderabad 500 059, India

## Abstract

Plant growth promoting rhizobacteria (PGPR) has been identified as a group of microbes that are used for plant growth enhancement and biocontrol for management of plant diseases. The inconsistency in performance of these bacteria from laboratory to field conditions is compounded due to the prevailing abiotic stresses in the field. Therefore, selection of bacterial strains with tolerance to abiotic stresses would benefit the end-user by successful establishment of the strain for showing desired effects. In this study we attempted to isolate and identify strains of *Bacillus* and *Pseudomonas* spp. with stress tolerance and proven ability to inhibit the growth of potential phytopathogenic fungi. Screening of bacterial strains for high temperature (50°C), salinity (7% NaCl), and drought (−1.2 MPa) showed that stress tolerance was pronounced less in *Pseudomonas* isolates than in *Bacillus* strains. The reason behind this could be the formation of endospores by *Bacillus* isolates. Tolerance to drought was high in *Pseudomonas* strains than the other two stresses. Three strains, P8, P20 and P21 showed both salinity and temperature tolerance. P59 strain possessed promising antagonistic activity and drought tolerance. The magnitude of antagonism shown by *Bacillus* isolates was also higher when compared to *Pseudomonas* strains. To conclude, identification of microbial candidate strains with stress tolerance and other added characteristic features would help the end-user obtain the desired beneficial effects.

## 1. Introduction

A wide range of agriculturally important microorganisms (AIMs) have been exploited for crop health management, which comprise nitrogen fixers like* Rhizobium*,* Bradyrhizobium*,* Sinorhizobium*,* Azotobacter*,* Azospirillum*, phosphate solubilisers like* Bacillus*,* Pseudomonas*,* Aspergillus, *and arbuscular mycorrhizae (AM); and fungi, bacteria, viruses and nematodes used for pest and disease management in agriculture, horticulture, and forestry. Plant growth promoting rhizomicroorganisms (PGPR) is known to increase plant growth and induce host plant resistance and crop yield [1].

As the crops are affected by abiotic stresses such as soil moisture deficit stress, high temperature, soil salinity, and so forth, microbes are also known to be affected by these conditions. Reports from Madhya Pradesh and Chhattisgarh of India indicated that the free living rhizobial population declines to lower than the minimum threshold levels required for nodulation due to high soil temperatures requiring inoculation every year [2, 3]. Widden and Hsu [4] observed that the ability of different species of* Trichoderma *to colonize pine or maple litter differed with temperatures.

*Bacillus*,* Pseudomonas, *and other microorganisms have been extensively studied for their ability to solubilize nutrients, their biocontrol potential, and their plant growth promoting abilities in all crop production systems. However, successful deployment of these organisms in stressed ecosystems depends on their ability to withstand and proliferate under adverse environments such as high temperatures, salt stress, mineral deficiency, heavy metal toxicity, and so forth. Inconsistency and variability in yield responses have also been attributed to adverse conditions such as interaction with other rhizospheric organisms, physical and chemical conditions of the soil (e.g., low pH), poor ability of the PGPR strain to colonize the plant roots, and environmental factors including high mean temperatures and low rainfall during the growing season [5].

A major problem in rainfed agroecosystems is predominance of abiotic stresses like high temperature, salinity, and drought where the survival of bioinoculants is a problematic issue. The variations in results from laboratory to field are more compounded due to various abiotic stresses that prevail under field conditions for a microbial inoculant to establish and to show the desired effect. Such problems can be overcome by sound screening programme for efficient stress tolerant PGPRs for effective deployment of these strains to draw one or more beneficial effects. Hence, the present study was conducted to identify strains of* Pseudomonas* and* Bacillus* collected from crop production systems from different agroecological regions of India for their ability to withstand adverse environments such as high temperature, salinity, and drought along with antagonistic activity.

## 2. Materials and Methods

### 2.1. Soil Samples and Soil Characteristics

Seventy-five (75) bulk, rhizosphere and nonrhizosphere soil samples of different crops representing 31 locations from 13 states of the country were obtained. For all soil samples, various geographical and environmental characters like agroecological region, climate, soil type, mean annual rainfall, and maximum soil temperature and physical characteristics like pH, EC, and particle size and chemical characters like NPK and OC parameters were determined following standard procedures [6].

### 2.2. Isolation of* Pseudomonas* and* Bacillus* spp

Soil samples were processed further for isolation of fluorescent* Pseudomonas* spp. using King's B medium [7] and* Bacillus* spp. were obtained using heat enrichment and dilution plating of soil samples on nutrient agar medium.

### 2.3. Screening for Antagonistic Activity

Maltose-dextrose agar was used for assessing the antagonistic activity of all isolates of* Pseudomonas *and* Bacillus* against major plant pathogens, namely,* Botrytis ricini*,* Fusarium oxysporum *f.sp.* ricini*,* Macrophomina phaseolina*,* Rhizoctonia solani*,  and* Sclerotium rolfsii*. Screening for antagonistic activity was followed by carrying the dual culture method as described by [8] for identifying potential isolates possessing antagonistic activity against test pathogens. Isolates inhibiting the growth of all test pathogenic fungi were further evaluated for their potential for fungal growth inhibition following bangle method as described below. Efficacy of 8 fluorescent* Pseudomonas *isolates was tested against the test pathogens by dual plate assay on petriplates containing maltodextrose agar using the bangle method where the bangle (70 mm dia) was dipped for 2 min in the culture of bacterial antagonist, multiplied in King's B broth and placed on the solidified medium in a petriplate. Five mm discs of pathogen cut from the periphery of the actively growing cultures were kept in the middle of the bangle. Control plates had only fungus. Petriplates were sealed with parafilm and incubated at 28 ± 2°C in a BOD incubator for 6 days. Radial growth of fungus was recorded and percent inhibition was calculated. Antagonistic activity was expressed as percent inhibition of fungal growth.

### 2.4. Screening of Isolates for Abiotic Stress Tolerance

Isolates were screened for their ability to tolerate different abiotic stresses (high temperature (50°C), salinity (1.2 M), and drought (−1.2 MPa) using tryptone soy broth (TSB). Growth of all isolates was recorded using spectrophotometer at 600 nm with uninoculated medium as blank. Bacterial isolates were considered stress tolerant if an OD of 0.1 was recorded.

#### 2.4.1. High Temperature Tolerance

Ten mL of TSB was dispensed into 30 mL screw cap tubes and autoclaved. Fresh cultures of test strains were grown for 6 h on a shaker incubator and the bacterial population was adjusted to 2 × 10^5^ per mL and used as initial inoculum. Inoculated tubes were incubated at 50°C for 24 h and OD was recorded.

#### 2.4.2. Salinity Tolerance

Ten mL of TSB amended with 7% NaCl was dispensed in 30 mL capacity screw cap tubes and autoclaved. Fresh cultures of test strains grown for 6 h on a shaker incubator were adjusted to 2 × 10^5^ per mL population and used as initial inoculum. The inoculated tubes were incubated at 28°C for 24 h and OD was recorded.

#### 2.4.3. Drought Tolerance

To characterize drought tolerance, a known quantity of TSB medium amended with 32.6% of polyethylene glycol-6000 (326 gm PEG per 1 L media creates an osmotic pressure of −1.2 Mpa) was dissolved by heating on a hot plate, and then the final volume was made up to 1 Lit with PEG unamended medium. The liquid medium was dispensed in 30 mL capacity screw cap tubes and autoclaved. Fresh cultures of test strains grown for 6 h on a shaker incubator were adjusted to 2 × 10^5^ per mL population and used as initial inoculum. The inoculated tubes were incubated at 28°C for 24 h and OD was recorded.

## 3. Results

### 3.1. Isolation of Bacteria

A total of 75 fluorescent* Pseudomonas* spp. and 120* Bacillus* spp. were isolated from soil samples obtained from 31 different locations representing 13 states of India. Isolates were designated as P1 to P75 and B1 to B120, respectively, and added to the culture collection of Central Research Institute for Dryland Agriculture, Hyderabad. All the isolates were stored as 30% glycerol stocks at −20°C and revived periodically for further studies.

### 3.2. Abiotic Stress Tolerance

All the isolates were screened for their ability to tolerate high temperature (50°C), salinity (1.2 M), and drought (−1.2 MPa)* in vitro*. Out of 75 isolates, seven (P8, P12, P14, P15, P20, P21, and P28) could tolerate 50°C, seven could tolerate salinity levels of 1.2 M (7% NaCl), namely, P8, P20, P21, P22, P37, P42, and P43 and 14 isolates (P6, P17, P30, P59, P60, P62, P64, P65, P67, P68, P69, P70, P73, and P74) tolerated −1.2 MPa osmotic stress. The ability of isolates along with their ability to tolerate these stresses is summarized in Table 1. Among the temperature tolerant isolates, P8 was isolated from Solapur, Maharashtra, whereas, P20, P21, and P22 were isolated from Hayathnagar, Hyderabad, Andhra Pradesh soils and P37 was isolated from Hisar, Haryana. P42 and P43 were isolated from Bhopal (Madhya Pradesh) and Rajkot (Gujarat), respectively. P8, P20, and P21 showed both temperature and salinity tolerance. Whereas, the remaining isolates showed tolerance to only one type of stress (Table 1).

**Figure 1:**
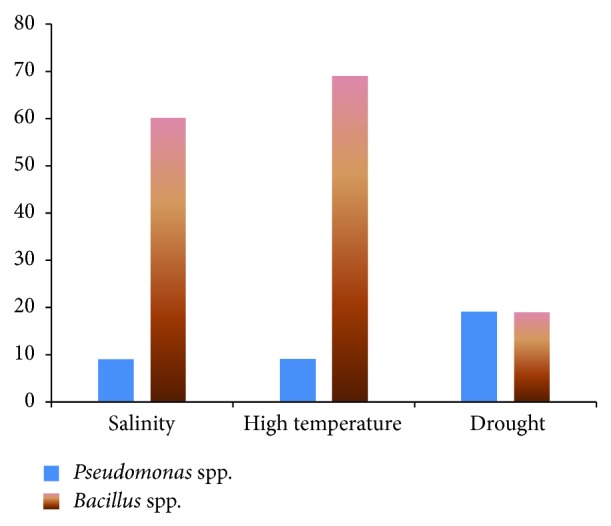
Percentage of* Pseudomonas* and* Bacillus* strains exhibiting various abiotic stresses.

Out of 120* Bacillus* isolates 23 strains could tolerate −1.2 MPa osmotic stress, 83 strains could tolerate 50°C, and 72 strains tolerated salinity levels of 1.2 M. The ability of isolates to tolerate these stresses is summarized in Table 1. Among the tested strains, the number of* Bacillus* spp. exhibiting different stresses was higher than that of* Pseudomonas* strains (Figure 1).

### 3.3. *In Vitro* Antagonistic Activity

In a preliminary assay, the dual culture method was followed to screen potential antagonistic strains. Out of 75* Pseudomonas* isolates tested, 16 could successfully inhibit growth of* Botrytis ricini*. Whereas nine isolates inhibited growth of* Fusarium oxysporum *f. sp.* ricini*,* Macrophomina phaseolina* was inhibited by 20 isolates. Eleven isolates inhibited growth of* Rhizoctonia solani* and eight isolates arrested growth of* Sclerotium rolfsii *(Table 2). Seven isolates, namely, P18, P19, P20, P21, P42, P43, and P59 inhibited growth of all the test phytopathogenic fungi. P17 could inhibit growth of* F. oxysporum* f. sp.* ricini* and* M. phaseolina *(Table 2). Out of 120* Bacillus* isolates evaluated, 49 isolates could inhibit the growth of* S. rolfsii *and* M. phaseolina*.* R. solani *was inhibited by 32 isolates, 51 isolates inhibited growth of* Fusarium oxysporum *f. sp.* Ricini,* and 30 isolates inhibited growth of* Botrytis ricini *(Table 3). Sixteen isolates could inhibit all five phytopathogens, namely, B1, B5, B9, B15, B18, B35, B43, B45, B64, B77, B81, B82, B87, B96, B101, and B120.

**Figure 2:**
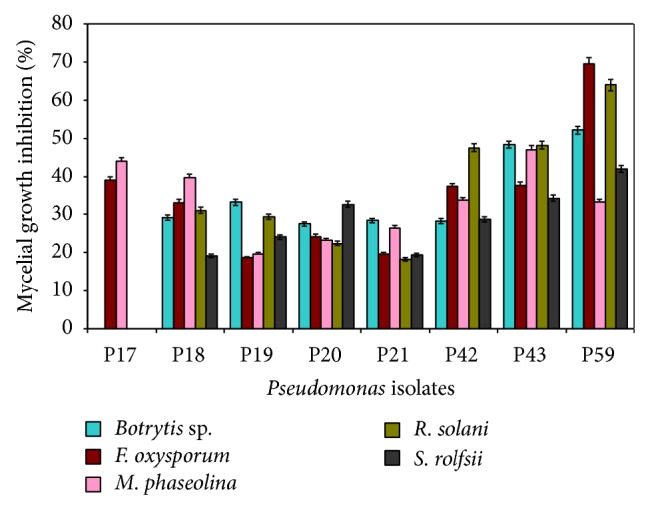
Antagonistic activity of selected* Pseudomonas* isolates against phytopathogenic fungi.

Further screening for antifungal activity using the bangle method revealed that P59 was highly antagonistic towards* B. ricini* showing an inhibition of 52.1% followed by P43 inhibiting 48.3% growth which did not differ significantly. Against* F. ricini* f. sp.* ricini* also P59 was very effective with an inhibition of 69.5% of growth and P17 was the next best isolate with 38.9% inhibition (Figure 2). P43 was the best isolate to inhibit growth of* M. phaseolina *showing 46.3% inhibition and P17 was the next best isolate (43.9%). P59 reduced growth of* R. solani* by 64% followed by P43 (48.2%). In the case of* S. rolfsii*, also, P59 was identified as the best isolate with growth by 41.9% followed by P43 which inhibited growth by 34.2% (Figure 2). Hence, P59 and P43 were identified as the best antagonistic strains. In the secondary screening, the bangle assay method was followed in order to have a clear idea of individual antagonistic strains against each phytopathogen. B77 inhibited 77.8% growth of* S. rolfsii *and B81 inhibited 58.9% growth of* Macrophomina phaseolina*. B120 inhibited growth of* Rhizoctoniasolani* by 80% (Table 4). B18 and B77 inhibited 66.7% growth of* Fusarium oxysporum* f. sp.* ricini*, whereas B82 has shown maximum inhibition of 61.1% against* Botrytis ricini *followed by B64 and B35 (57.8%).

## 4. Discussion

In view of the global climate change scenario, agriculture is one of the essential areas getting affected, ultimately showing impact on food productivity. Therefore, selection, screening, and application of stress tolerant PGPRs for improved farming would significantly help the farming community by overcoming such drastic climate changes. Further, such PGPR application is also known to overcome the deleterious effect of chemical fertilizers and pesticides. Therefore, an attempt was made to fish-out and identify promising bacterial isolates of* Bacillus* and* Pseudomonas* spp. with abiotic stress tolerance and antagonistic activity for better plant growth promotion.

Survival of an introduced strain in the rhizosphere is affected by a number of abiotic factors such as high salt, high pH, and high temperature [9, 10]. An understanding of the ability of* Pseudomonas *and* Bacillus *spp. isolated from various agroecosystems of India to withstand different abiotic stresses will enable an appropriate deployment of such strains for deriving beneficial effects. Therefore, all the isolates in the current work were evaluated for their ability to tolerate abiotic stresses.

Among the tested* Pseudomonas* isolates for stress tolerance seven isolates were found to possess salinity tolerance. Johri et al. [10] isolated and characterized salinity tolerant phosphate solubilizing bacteria that could survive at 5% NaCl concentration. Recently, Tank and Saraf [11] reported the plant growth promoting effect of* P. fluorescens* and* P. aeruginosa* on tomato and these strains were able to survive at 6% NaCl. Paul et al. [12] explained that* Pseudomonas fluorescens* strain MSP-393 synthesized novel proteins which nullified detrimental effects of high osmolarity. Salt stress tolerance is an important aspect of saprophytic ability and competitiveness among rhizobial isolates [13]. Therefore, it could be observed from the current results that some of the salt tolerant isolates may have good saprophytic and competitive abilities to perform well in the rhizosphere.

Seven isolates were identified with high temperature tolerance. Ali et al. [14] reported a strain of* Pseudomonas* AKM-P6 that enhanced tolerance of sorghum seedlings in high temperature and tolerance of the reported strain was due to synthesis of heat shock proteins. Isolates P8, P20, and P21 exhibited both salinity and temperature tolerance (Table 1). Tolerance to drought was more pronounced in* Pseudomonas* strains in the current case compared to other stresses. Rehman and Nautiyal [15] reported a drought tolerant* Rhizobium* sp. which could survive 45% of PEG concentration. In the current experiment, the P17 strain with good plant growth promoting ability survived 40.5% PEG concentration (data not shown). Timmusk and Wagner [16] demonstrated that inoculation with* Paenibacillus polymyxa* protected* Arabidopsis thaliana* from drought stress by increasing the expression of the stress-induced gene Erd15. The formation of exopolysaccharides (EPSs) by rhizosphere bacteria is one of the important mechanisms in exerting drought tolerance. The EPS produced in turn plays an important role in soil aggregation thereby improving soil water holding capacity and fertility [17, 18]. High temperature, salinity, and drought tolerance was more pronounced in* Bacillus* strains compared to* Pseudomonas* isolates (Figure 1). This could be due to an endospore forming capacity of* Bacillus* spp.

In case of* Bacillus* spp., the number of isolates showing tolerance to said abiotic stresses was relatively high compared to* Pseudomonas* isolates (Table 1). This could be due to endospore formation in* Bacillus* spp. Piggot and Hilbert [19] highlighted that Bacillus endospores are extremely resistant dormant forms capable of withstanding unfavorable environmental conditions. Such spore forming, stress tolerant* Bacillus* isolates find application development of powder formulations as their population can be maintained at desired levels. Spore formation by selected PGPRs also offers an advantage, since they usually have higher shelf-life after postculture conditioning as bacterial suspensions and powder formulations [20].

The reason behind the evaluation of abiotic stress tolerance among the isolated strains was that these stress tolerant strains can be efficiently deployed in extreme environments where they can show better rhizosphere competence and saprophytic competitive ability. Interestingly, some of the abiotic stress tolerant strains also protected plants from abiotic stresses like drought [16], chilling injury [21], high temperature [14], and salinity [22].

*Pseudomonas* spp. are well known biocontrol agents used for the control of soil-borne phytopathogenic fungi. Various mechanisms have been attributed to their antagonistic activity, namely, different hydrolytic enzymes, chitinases, HCN, and siderophore production and production of antibiotics like phenazines, DAPG, pyrrolnitrin, pyoluteorin, and so forth. Some of these mechanisms in turn also make* Bacillus* spp. an ideal biocontrol agent. In the current study,* Pseudomonas *isolates P43 and P59 inhibited the growth of all the test phytopathogenic fungi effectively. The said mechanisms were evaluated in the strains of the current study to identify the various reasons for antagonism [23]. In turn, P59 that inhibited the growth of all five fungi also possessed drought tolerance (Table 1). This feature of possessing both characters makes the selection an ideal one for their better performance under field conditions. This feature of exhibiting antagonism, more pronounced in* Bacillus* isolates than* Pseudomonas* spp. Pseudomonads represent the major group of nondifferentiating microorganisms that produce antibiotics such as phycocyanin and pyrrolnitrin, and pseudomonic acid was investigated* in vitro* and* in vivo* by Kaleli et al. [24]. In the current study,* Pseudomonas* strains P42, P43, and P59 inhibited the growth of all five pathogenic fungi, among which P42 and P43 were salinity tolerant and whereas P59 strain was tolerant to drought. However, such commonality was least identified in* Bacillus* isolates except B77 which potentially inhibited the growth of two fungi and exhibited tolerance to stresses.

## 5. Conclusion

Within the present scenario, the most well studied phenomenon is the antagonistic activity of rhizosphere microorganisms towards plant pathogens with the resultant suppression of plant disease. However, a major problem with such biological agents is the inconsistency in field performance, which is not only the net outcome of complex interactions involving plant, biological agents, pathogen, and the physical and biological environments but also attributed to their poor rhizospheric competence; therefore, a careful choice of different conditions is necessary if meaningful data are to be generated. This study therefore paves the way for the ideal selection of bioagents having abiotic stress tolerance and proven antagonistic activity for their consistent performance under field conditions. Further, strains of* Pseudomonas* (P42, P43, and P59) and* Bacillus* (B77) possessing tolerance to abiotic stress (es) and antagonistic activity would enlist them as candidate strains for further characterization and application.

## Figures and Tables

**Table 1:** List of *Pseudomonas* and *Bacillus* isolates showing tolerance to various abiotic stresses.

	*Pseudomonas* isolates showing tolerance to		
	High temperature (50°C)	Salinity (14 × 10^2^ ** **dS/m)	Drought (−1.2** **MPa)
*Pseudomonas* spp.	P8, P12, P14, P15, P20, P21, and P28 (7 strains)	P8, P20, P21, P22, P37, P42, and P43 (7 strains)	P6, P17, P30, P59, P60, P62, P64, P65, P67, P68, P69, P70, P73, and P74 (14 strains)

*Bacillus* spp.	B1 to B3, B5 to B22, B24, B25, B27 to B29, B31 to B33, B35, B37, B39–B44, B46 to 49, B51 to B59, B61, B62, B64, B65, B66 to B68, B70, B72 to B79, B83, B85, B86, B88, B93, B98, B99, B100, B103, B105, B112, B114 to B117, B119, and B120 (83 strains)	B2, B3, B5 to B9, B11, B12, B14, B17 to B24, B28, B29, B31, B32, B35, B36, B38, B39, B40, B42, B44 to B46, B53 to B55, B57, B61, B65, B72 to B75, B77 to B80, B82 to B91, B93, B97 to B100, B102 to B105, B106 to B108, B112, B114, B116, B117, and B120 (72 strains)	B16, B17, B38, B39, B47, B49, B53 to B55, B57, B58, B60, B61, B64, B66, B69, B73, B84, B90, B92, B93, B98, and B105 (23 strains)

**Table 2:** Antagonistic activity of *Pseudomonas* isolates against phytopathogenic fungi.

*Botrytis ricini *	*Fusarium oxysporum* f. sp. *ricini *	*Macrophomina phaseolina *	*Rhizoctonia solani *	*Sclerotium rolfsii *
P18, P19, P20, P21, P41, P42, P43, P46, P47, P51, P55, P57, P58, P59, P66, and P67 (16 strains)	P17, P18, P19, P20, P21, P41, P42, and P43, P59 (9 strains)	P3, P6, P7, P10, P17, P18, P19, P20, P21, P25, P28, P29, P33, P39, P42, P43, P49, P59, P63, and P70 (20 strains)	P18, P19, P20, P21, P42, P43, P49, P51, P53, P59, and P62 (11 strains)	P18, P19, P20, P21, P39, P42, P43, and P59 (8 strains)

**Table 3:** Antagonistic activity of *Bacillus* isolates against phytopathogenic fungi.

*Sclerotium rolfsii *	*Macrophomina phaseolina *	*Rhizoctonia solani *	*Fusarium oxysporum* f. sp. *ricini *	*Botrytis ricini *
B1, B5, B9, B11, B12, B15, B16, B18 to B21, B23, B24, B29, B30, B32, B35 to B40, B43, B45, B47, B49, B64, B66, B72, B73, B75 to B79, B81, B82, B86 to B88, B90, B92 to B96, B101, B111, and B120 (49 isolates)	B1, B5, B9, B12, B15, B16, B18, B20, B21, B23, B25, B27, B29, B33, B35 to B37, B42 to B45, B47, B61, B64 to B66, B72, B73, B75 to B79, B81, B82, B86 to B88, B90, B92 to B96, B99, B101, B103, B111, and B120 (49 isolates)	B1, B5, B8, B9, B11, B15, B18, B23, B30, B32, B35, B36, B38, B43, B45, B47, B61, B64, B75, B77, B81, B82, B86 to B88, B90, B96, B99, B101, B103, B111, and B120 (32 isolates)	B1,B5, B8, B9, B11, B12, B15, B16, B18, B19, B21, B23, B24, B25, B29, B30, B32, B35, B37, B38, B42 to B46, B55, B56, B61, B64, B66, B67, B71 to B73, B75, B77, B81, B82, B86 to B88, B92, B93, B95, B96, B99, B101, B103, B108, B111, and B120 (51 isolates)	B1, B5, B9, B15, B18, B22, B35, B36, B42 to B47, B55, B56, B61, B64, B66, B67, B71, B77, B81, B82, B87, B93, B96, B101, B103, and B120 (30 isolates)

**Table 4:** Percentage of inhibition of pathogen by antagonistic *Bacillus* by bangle method.

Strains	*Sclerotium rolsfii *	*Macrophomina phaseolina *	*Rhizoctania solani *	*Fusarium *sp.	*Botrytis* sp.
B1	75.6	50.0	26.8	25.1	53.3
B5	75.6	53.3	29.4	61.1	52.2
B9	73.3	52.2	31.5	64.4	50.0
B15	73.3	52.2	29.1	57.8	50.0
B18	72.2	35.0	33.0	66.7	52.2
B35	72.2	52.2	35.2	61.1	57.8
B43	36.2	38.3	41.5	62.2	55.6
B45	66.7	40.4	65.6	57.8	53.3
B64	72.2	36.2	40.0	55.6	57.8
B77	75.8	57.8	64.4	66.7	55.6
B81	72.2	58.9	55.6	58.9	55.6
B82	75.6	56.7	66.7	61.1	61.1
B87	73.3	55.6	68.9	58.9	46.7
B96	75.6	50.0	66.7	58.9	41.4
B101	75.6	55.6	66.7	61.1	55.6
B120	71.1	57.8	80.0	62.2	55.6
